# Molecular elucidation of a new allelic variation at the *Sg-5* gene associated with the absence of group A saponins in wild soybean

**DOI:** 10.1371/journal.pone.0192150

**Published:** 2018-01-30

**Authors:** Jagadeesh Sundaramoorthy, Gyu Tae Park, Kyosuke Mukaiyama, Chigen Tsukamoto, Jeong Ho Chang, Jeong-Dong Lee, Jeong Hoe Kim, Hak Soo Seo, Jong Tae Song

**Affiliations:** 1 School of Applied Biosciences, Kyungpook National University, Daegu, Republic of Korea; 2 Faculty of Agriculture, Iwate University, Morioka, Iwate, Japan; 3 Department of Biology Education, Kyungpook National University, Daegu, Republic of Korea; 4 Department of Biology, Kyungpook National University, Daegu, Republic of Korea; 5 Department of Plant Science, Seoul National University, Seoul, Republic of Korea; College of Agricultural Sciences, UNITED STATES

## Abstract

In soybean, triterpenoid saponin is one of the major secondary metabolites and is further classified into group A and DDMP saponins. Although they have known health benefits for humans and animals, acetylation of group A saponins causes bitterness and gives an astringent taste to soy products. Therefore, several studies are being conducted to eliminate acetylated group A saponins. Previous studies have isolated and characterized the *Sg-5* (*Glyma*.*15g243300*) gene, which encodes the cytochrome P450 72A69 enzyme and is responsible for soyasapogenol A biosynthesis. In this study, we elucidated the molecular identity of a novel mutant of *Glycine soja*, ′CWS5095′. Phenotypic analysis using TLC and LC-PDA/MS/MS showed that the mutant ′CWS5095′ did not produce any group A saponins. Segregation analysis showed that the absence of group A saponins is controlled by a single recessive allele. The locus was mapped on chromosome 15 (4.3 Mb) between Affx-89193969 and Affx-89134397 where the previously identified *Glyma*.*15g243300* gene is positioned. Sequence analysis of the coding region for the *Glyma*.*15g243300* gene revealed the presence of four SNPs in ′CWS5095′ compared to the control lines. One of these four SNPs (G1127A) leads to the amino acid change Arg376Lys in the EXXR motif, which is invariably conserved among the CYP450 superfamily proteins. Co-segregation analysis showed that the missense mutation (Arg376Lys) was tightly linked with the absence of group A saponins in ′CWS5095′. Even though Arg and Lys have similar chemical features, the 3D modelled protein structure indicates that the replacement of Arg with Lys may cause a loss-of-function of the Sg-5 protein by inhibiting the stable binding of a heme cofactor to the CYP72A69 apoenzyme.

## Introduction

Saponins are glycosylated compounds that are widely distributed in plants, and have different biological and pharmaceutical properties [1,2]. In addition, saponins are structurally diverse, for example triterpenoid and steroidal saponins [3]. Triterpenoid saponins are widely distributed in higher plants [1,2]. They are composed of triterpene aglycone, with one or more sugar chains. Biosynthesis of saponins is initialized from isopentenyl pyrophosphate in the mevalonate pathway [3]. Triterpene aglycones are derived from the 30-carbon linear 2,3-oxidosqualene precursor. In the first step of saponin synthesis, 2,3-oxidosqualene is cyclized by oxidosqualene cyclases (OSCs) to produce polycyclic triterpene [4,5]. After cyclization of the basic triterpene backbone, the backbone is oxidized to produce a hydrophobic aglycone called sapogenin. The oxidization step is catalyzed by cytochrome P450 (CYP450s) mono-oxygenases [6]. The next step is to synthesize saponins via *O*-glycosylation of the aglycones. Studies on triterpenoid saponins have been undertaken on crops and medicinal plants due to their important commercial uses in cosmetic and pharmaceutical industries. However, the biological roles of triterpenoid saponins in plants remain underexplored.

Soybean seeds are abundant in high-quality proteins and fats. In addition, triterpenoid saponins are the major components of mature seeds [7,8]. Soybean seeds on average contain 0.2–0.5% of saponin, although this value can widely vary depending on cultivars, degree of maturity, and growing locations [9–11]. In the well-known medicinal plant *Panax ginseng*, saponins are abundant in roots [12]. However, soybean saponins are rich in hypocotyls rather than in cotyledons and other plant parts [13,14]. Szakiel et al. [15] reported that oleanolic acid glycoside, a type of saponins in *Calendula officinalis* were synthesized in the cytoplasm and subsequently transported through the vacuolar membrane and finally accumulated in the vacuole. However, the site of saponin biosynthesis in soybean is still unknown.

**Fig 1:**
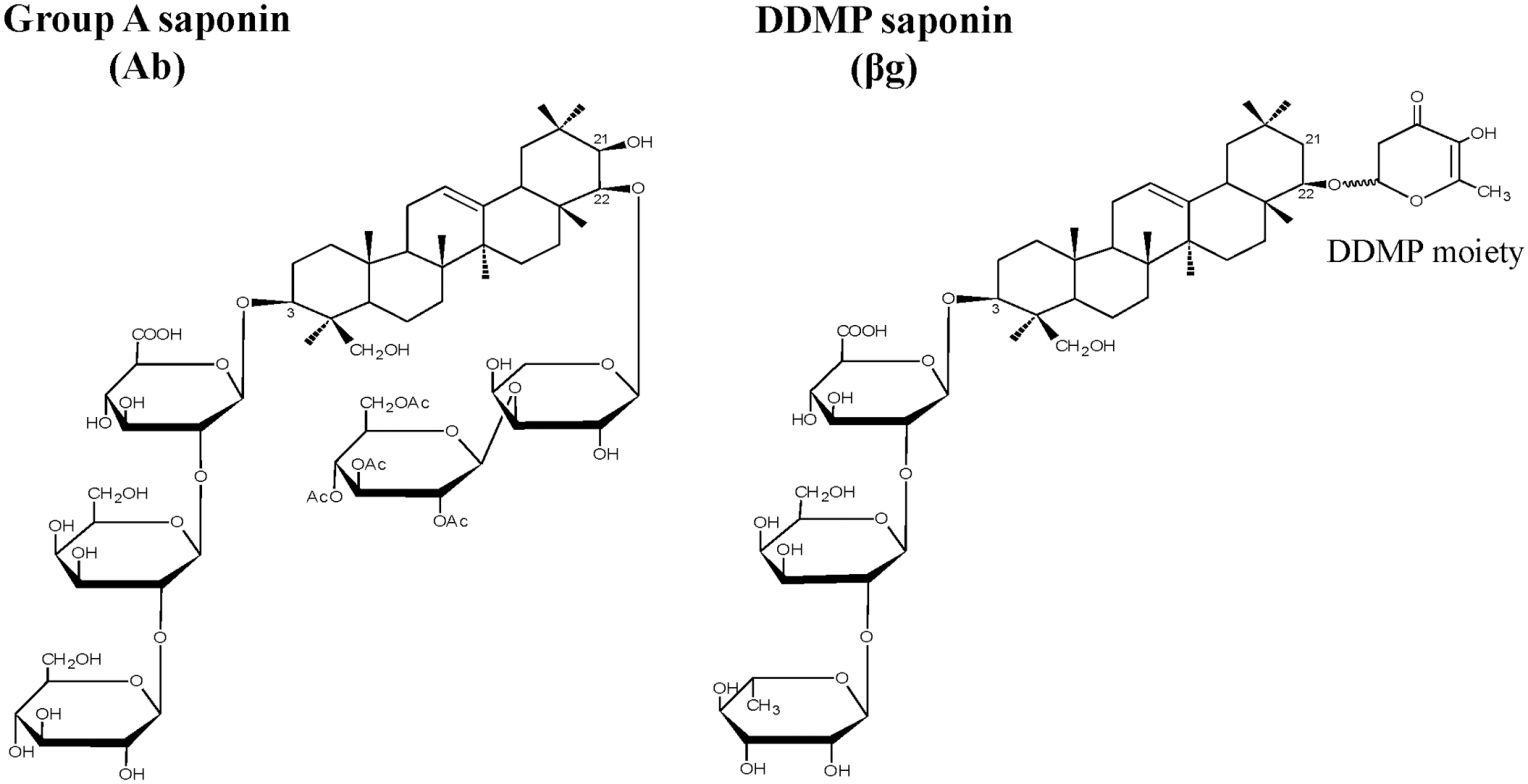
Chemical structure representation of group A (Ab) and DDMP (βg) triterpenoid saponins. The group A saponin (Ab) is bisdesmoside glycoside that contains two sugar chains attached at the C-3 and C-22 hydroxyl positions of soyasapogenol A. The DDMP saponin (βg) is monodesmoside glycoside that contains a single sugar chain attached to the C-3 and the 2,3-dihydro-2,5-dihydroxy-6-methyl-4H-pyran-4-one (DDMP) moiety at the C-22 hydroxyl positions of soyasapogenol B.

Soybean saponins are mainly classified into group A and 2,3-dihydro-2,5-dihydroxy-6-methyl-4H-pyran-4-one (DDMP) saponins, which are commonly known as bisdemoside and monodesmoside saponins, respectively. Group A saponins contain two sugar chains at the C-3 and C-22 hydroxyl groups on the triterpenoid aglycone (soyasapogenol A; 3β, 21β, 22β, 24-tetrahydroxyolean-12-ene) (Fig 1) [10,16,17]. DDMP saponins contain a single sugar chain at the C-3 hydroxyl position of aglycone (soyasapogenol B; 3β, 22β, 24-trihydroxyolean-12-ene) and a DDMP moiety at the C-22 position (Fig 1) [10,16,17]. DDMP saponins are unstable molecules, which then degrade during food processing and the saponin extraction process into group B and group E saponins after hydrolysis (S1 Fig) [18]. In addition, the C-21 hydroxyl group is specific to soyasapogenol A, which distinguishes it from soyasapogenol B (Fig 1).

Diverse health benefits of soybean saponins have been reported. Saponins showed the antiviral activity against HIV, antioxidant activity, cholesterol-lowering activity, growth inhibition of tumor cell lines and anticancer activity through their anti-inflammatory activity [19–23]. Although soybean saponins are well known for their health benefits, the terminal acetylated sugar at the C-22 position of group A saponins in soybean seeds causes bitterness and astringent taste in soy products [7,24]. Therefore, several studies have been focused on removing the astringent taste of soy foods products through genetic identification and modification of genes involved in the biosynthesis of saponins [8,17,25,26].

In previous studies, many UDP-glycosyltransferase (UGT) genes, namely the *Sg-1*, *Sg-3*, *Sg-4*, *GmSGT2*, and *GmSGT3* involved in soybean saponin biosynthesis, have been identified [7,16,27–31]. Cytochrome P450 (CYP) 72A subfamily proteins are involved in the legume triterpenoid saponin biosynthesis [32]. Very recently, a hydroxylase gene, *Sg-5* (*Glyma*.*15g243300*; previously annotated as *Glyma15g39090* in the Phytozome database v1.1), that encodes the CYP72A69 enzyme, has been characterized in soybean [17]. The natural mutation was first identified in wild soybean, ′B01082′, which does not accumulate soyasapogenol A; therefore, biosynthesis of group A saponins does not occur [8,26]. The *sg-5* mutant in ′B01082′ was found to have a premature stop codon (L164*) in *Glyma*.*15g243300* [17]. Yano et al. [17] developed two induced mutations (ENT-1376, R44* and ENT-1339, S348P) in *Glyma*.*15g243300* that lack or contain 4-fold lower concentration of group A saponins compared to the wild-type, respectively.

Krishnamurthy et al. [33] discovered a new mutant (′CWS5095′) that contained no group A saponins among 3025 wild soybean accessions collected from nine regions of Korea. This study aimed to determine the molecular basis of a new genetic component influencing the biosynthesis of group A saponins in the new mutant, ′CWS5095′. Analysis of saponin contents in both seed hypocotyl and cotyledon showed the complete absence of group A saponins in ′CWS50595′. We found that the biosynthesis of group A saponins in ′CWS5095′ is regulated by a single recessive allele of the *Sg-5* gene, designated *sg-5a*. Among the four SNPs detected in *sg-5a*, an SNP led to the unique amino acid change, R376K, in the EXXR motif, which is absolutely conserved among CYP450s in the plant kingdom. Even though Arg and Lys possess similar chemical feature, the 3D modelled protein structure showed that the mutation R376K may inhibit the stable binding of the heme cofactor and subsequently leads to loss-of-function of Sg-5 in the ′CWS5095′ mutant. This suggests that that EXXR motif is very essential for the functional activity of the CYP450s, especially in plants.

## Results

### Phenotypic and genetic elucidation of a new *G*. *soja* mutant that lacks group A saponins

**Fig 2:**
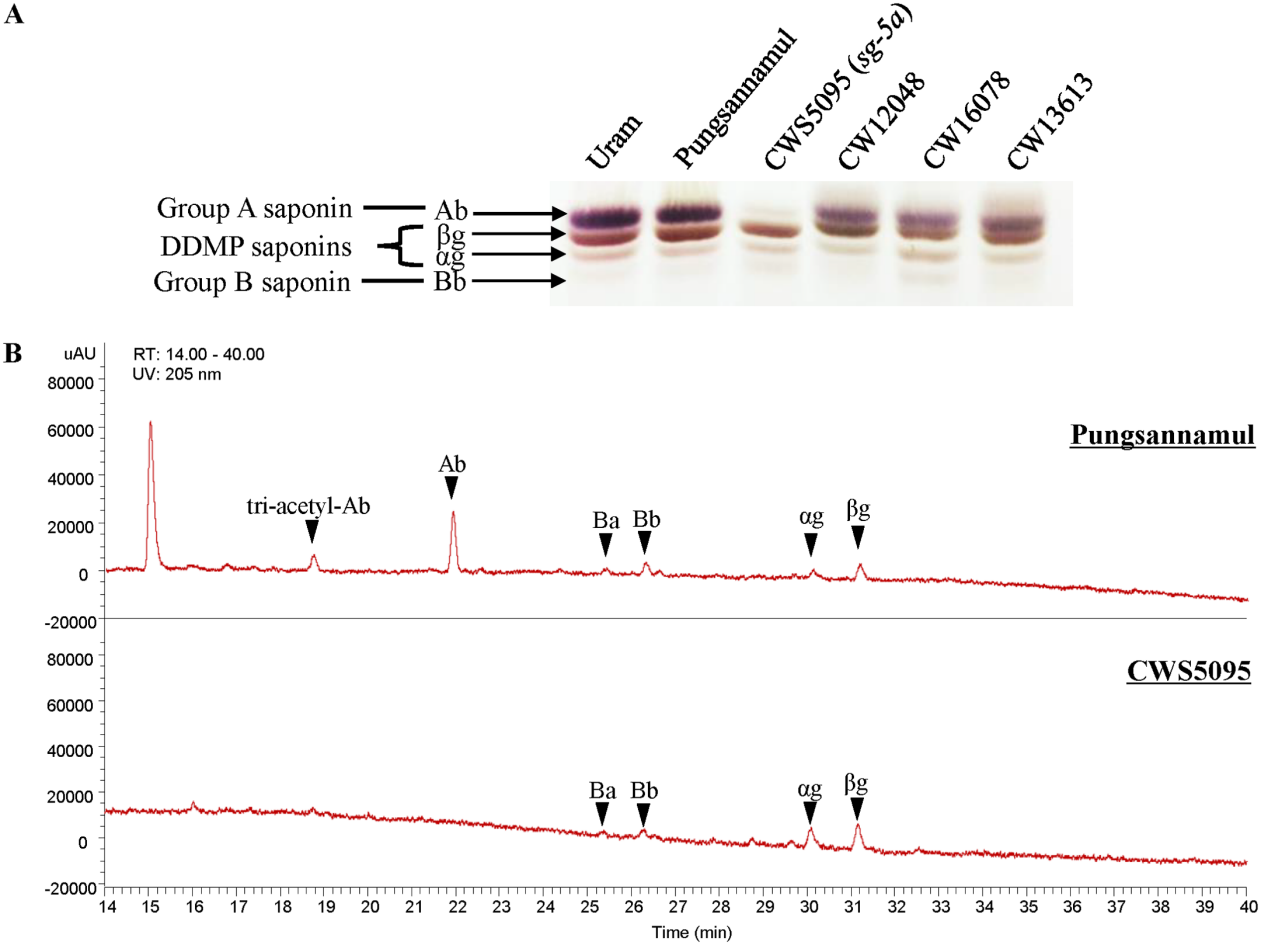
Separation and detection of saponins in seed hypocotyls. (A) The saponin phenotypes of wild and cultivated soybean accessions, along with new mutant, ′CWS5095′ in hypocotyl extracts (in 80% aqueous methanol), were detected by thin layer chromatography (TLC). (B) Seed hypocotyl extracts (in 80% aqueous methanol) of ′Pungsannamul′ (wild-type) and ′CWS5095′ (mutant) were separated by high-performance liquid chromatography (HPLC) and detected by UV absorption at 205 nm. The chemical structures of saponins corresponding to each peak were identified using LC-PDA/MS/MS. The chemical structures and corresponding names of the saponins are shown in Fig 1 and S1 Fig.

The wild soybean accession from Korea, ′CWS5095′, which does not contain any group A saponins, was discovered by Krishnamurthy et al. [33]. In the present study, we confirmed the phenotype of the mutant ′CWS5095′ by chromatography. The thin layer chromatography (TLC) patterns of the saponins in the hypocotyl extracts of ′CWS5095′ lacked group A saponins when compared to two cultivated (′Pungsannamul′ and ′Uram′) and three common wild (′CW12048′, ′CW16078′, and ′CW13613′) soybeans (Fig 2A). The LC-photodiode array (PDA)/MS/MS analysis also revealed that the hypocotyl extracts of ′CWS5095′ lacked peaks with the retention time expected for group A saponins, compared to cultivar ′Pungsannamul′ (Fig 2B). Furthermore, cotyledon extracts of ′CWS5095′ and ′Pungsannamul′ were also analyzed by LC-PDA/MS/MS and showed that group A saponins were absent in ′CWS5095′ (S2 Fig). These results confirm that ′CWS5095′ did not produce group A saponins.

For segregation analysis, crosses were performed between two common cultivars (′Pungsannamul′ and ′Uram′) and ′CWS5095′. A total of 120 F_2_ plants from the cross between ′Uram′ and ′CWS5095′ was segregated into 96 individuals with group A saponins and 24 without group A saponins (Table 1). The segregation fitted a 3:1 ratio. A similar pattern of segregation was observed in the population of the F_3_ progeny (from F_2_ heterozygous plants) derived from the cross between ′Pungsannamul′ and ′CWS5095′ (Table 1). The segregation analysis indicated that a single recessive allele controlled the mutant phenotype in ′CWS5095′.

**Table 1:** Segregation and co-segregation of F_2_ individuals for group A saponin phenotypes on soybean hypocotyl.

Cross	No. of F_2_ plants	F_3_ population (from F_2_ heterozygous plants)	Group A saponin phenotype					Genotype					
			Present	Absent	Expected	χ^2^	*p* value†	W*	H*	M*	Expected	χ^2^	*p* value†
Pungsannamul x CWS5095	-	304	233	71	3:1	0.22	**0.63**	71	162	71	1:2:1	0.65	**0.72**
Uram x CWS5095	120	-	96	24	3:1	0.86	**0.35**	29	67	24	1:2:1	1.07	**0.58**

^**†**^Not significant (*p* value>0.05)

*W: wild homozygote, H: heterozygote, M: mutant homozygote.

### Physical mapping of the *Sg-5* locus

We performed linkage analysis using an Affymetrix Axiom^®^ SNP array to determine the gene involved in soyasapogenol A biosynthesis in mutant ′CWS5095′. A physical map was constructed using 20 F_2_ individual lines derived from crosses between ′Pungsannamul′ and ′CWS5095′, and between ′Uram′ and ′CWS5095′. The locus was mapped to a 4.3 Mb region between the Affx-89193969 and Affx-89134397 SNP arrays on chromosome 15 (E) (S3 Fig). In a previous study, the *Sg-5* locus that controls the presence/absence of group A saponins in soybean was mapped genetically; it was found to be 1.2 cM distant from the SSR marker Satt117 on chromosome 15 (E) [8]. Yano et al. [17] characterized the *Sg-5* gene (*Glyma*.*15g243300*). The previously identified *Sg-5* locus (*Glyma*.*15g243300*) was located in the region that we mapped.

### Molecular analysis of the *sg-5a* allele

**Fig 3:**
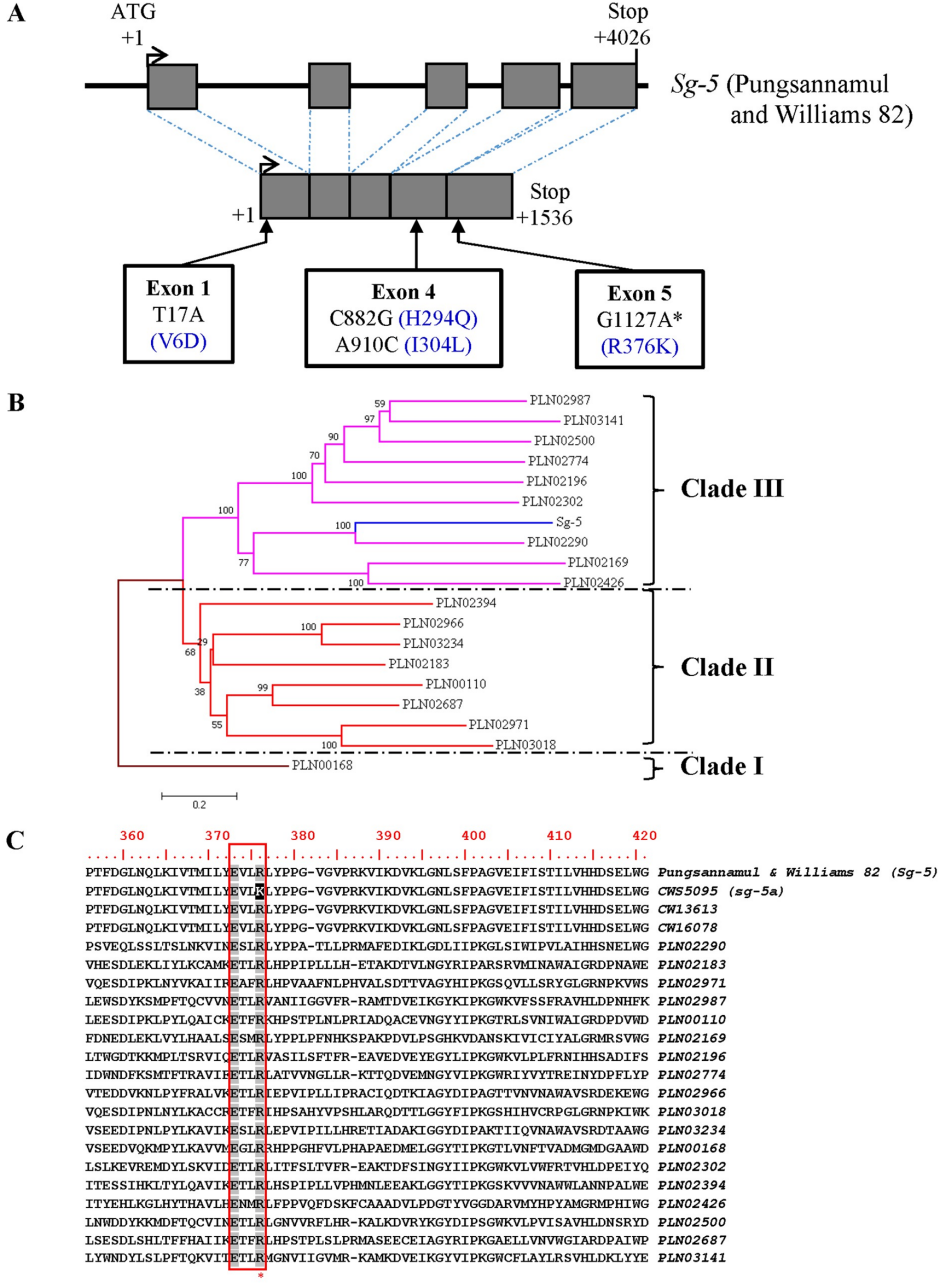
Gene structure of *Sg-5* (*Glyma*.*15g243300*) and the polymorphisms between ′Pungsannamul′ and ′CWS5095′. (A) Mutational changes in the recessive alleles at the *Sg-5* locus are indicated in the mutant by the white boxes. Exons are indicated by gray boxes. The asterisk indicates the most influential SNP out of the four SNPs detected in ′CWS5095′. (B) Phylogenetic tree constructed using neighbor-joining clustering of 18 CYP450 superfamily proteins belong to different plant species. CYP450 superfamily proteins were classified into three groups (clades I to III). The Sg-5 protein was positioned in clade III and was distantly related to the clade I CYP450 superfamily. (C) Comparison of amino acid sequence Sg-5 proteins with 18 CYP450 superfamily proteins from plants (GenBank accession numbers are provided in S4 Fig). An important amino acid substitution detected in ′CWS5095′ is highlighted in black and its position is marked with an asterisk. The EXXR motif is denoted by a red box and identical amino acids are shown in gray.

We analyzed coding sequence for *Glyma*.*15g243300* (position +1 to 1536; GenBank accession number MF624839) to determine whether *Glyma*.*15g243300* is responsible for the absence of group A saponins in the ′CWS5095′ mutant. The sequence analysis revealed that ′CWS5095′ contained four single-nucleotide polymorphisms (SNPs) compared to the common cultivars ′Pungsannamul′ and ′Williams 82′ (Fig 3A). The first SNP (T17A), detected in the first exon, converted valine into an aspartic acid (V6D). The second (C882G) and third (A910C) SNPs in the fourth exon led to a glutamine substitution for histidine (H294Q) and a leucine substitution for isoleucine (I304L), respectively. The fourth SNP (G1127A) was identified in the fifth exon and converted arginine into lysine (R376K).

Among the four SNPs detected in ′CWS5095′, the second and third SNPs were also detected in the coding sequences of two common *G*. *soja* accessions that contained group A saponins (′CW13613′ and ′CW16078′) (S4 Fig). This indicated that, among the four SNPs, the first and fourth were important mutations that might be involved in the allelic variation seen in ′CWS5095′.

CYP450s are mono-oxygenase hemoproteins that contain heme as a cofactor. In the NCBI- Conserved Domain Database (NCBI-CDD) database, CYP450 family proteins are clustered into 29 superfamilies based on the conserved domain models that generate overlapping annotations on the same protein sequence (https://www.ncbi.nlm.nih.gov/cdd). The protein sequences of 18 superfamilies registered for plant species were obtained from the NCBI-CDD database and were used to construct a phylogenetic tree (Fig 3B). Phylogenetic analysis showed that the Sg-5 protein belongs to clade III and is distantly related to the clades I and II CYP450 superfamily proteins. We performed multiple alignment analysis of the Sg-5 protein, along with the 18 CYP450 superfamilies in all three clades, to investigate the impact of the single-nucleotide substitutions on the allelic variations in ′CWS5095′ (Fig 3C and S4 Fig). The results of the multiple alignments showed that the first amino acid change (V6D) was not conserved among the CYP450 superfamily proteins (S4 Fig). However, the fourth amino acid substitution (R376K) in the EXXR motif was absolutely conserved among the CYP450 family proteins in plants, animals, fungi, and bacteria (Fig 3C) [34] with a few rare exceptions in some microorganisms [35,36]. The EXXR motif is known to be involved in heme binding and the stabilization of the tertiary structure of CYP450 proteins [37].

**Fig 4:**
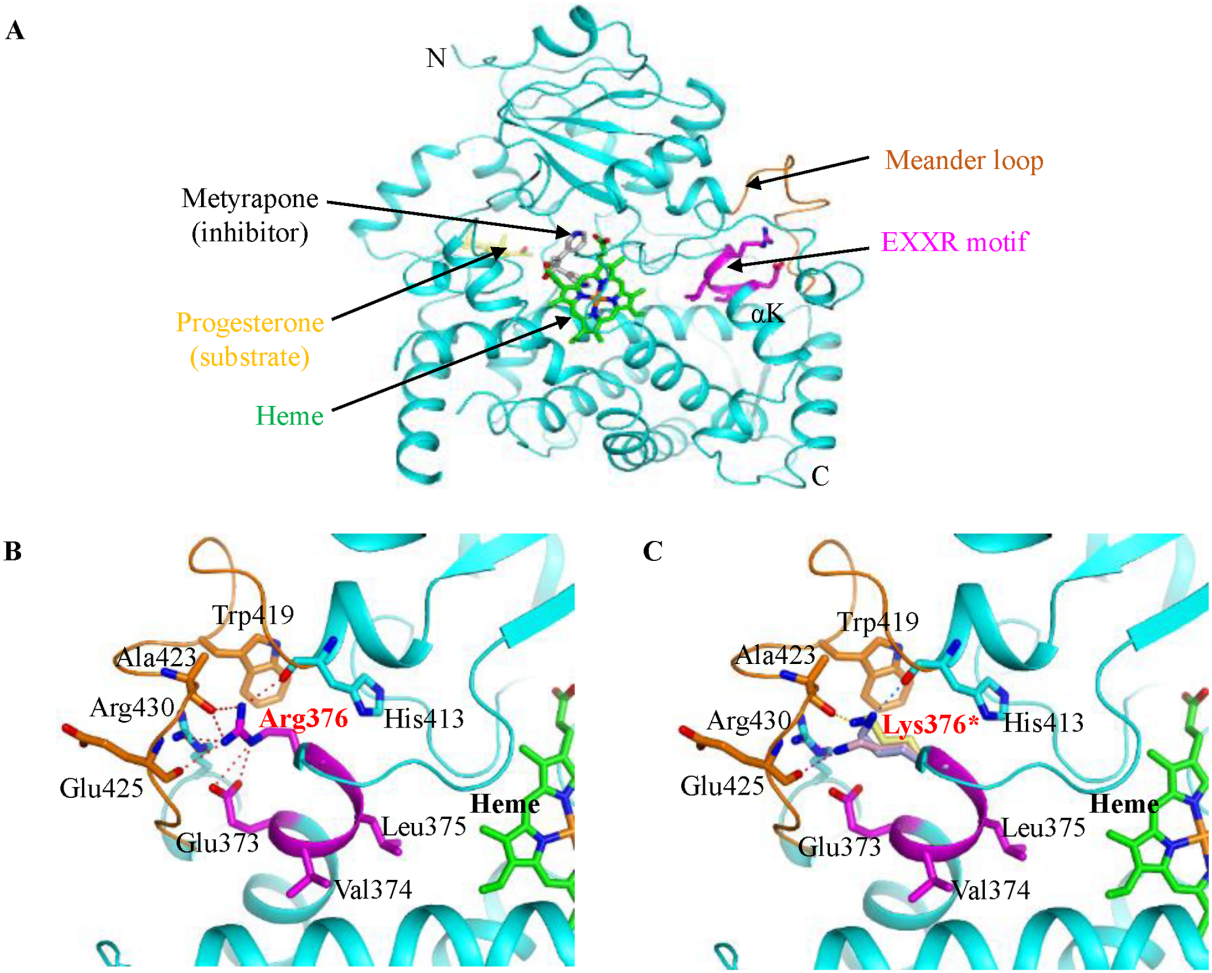
Three-dimensional model simulation of the amino acid substitution in the Sg-5 protein structure. (A) 3D model structure of the Sg-5 protein showing the heme cofactor (green), meander loop (brown), the EXXR motif (pink), and the αK helix that contains the EXXR motif and stabilizes heme by hydrophobic interactions. (B) Detailed view of wild-type Sg-5 protein showing that Arg376 in the EXXR motif interacts extensively with residues in the meander loop. Hydrogen bonds are shown in the side chains (red) of Glu373 and Arg430 and the main chains (red) of His413, Ala423, and Glu425. Arg376 also makes a hydrophobic interaction with the side chain of Trp419. (C) Detailed view of the mutant sg-5 protein from ′CWS5095′ showing the three rotamers of Lys376 and their corresponding hydrogen bonds in pink, light blue, and light yellow.

We performed a 3D model of Sg-5 using Phyre2, a web-based 3D structure modeling program [38] to obtain further insights into the effect of the noteworthy amino acid substitutions on Sg-5 protein function. The highest score in the 3D model output for Sg-5 suggested that the crystal structure of human CYP450 3a4 was the most appropriate model protein (PDP id: 1W0E_A) [39]. Based on the structures of apo and either the substrate or inhibitor complexes, the 3D model was able to show the binding sites of the substrate, inhibitor, a cofactor heme, and the EXXR motif (Fig 4A). The detailed view of the 3D model indicated that the Arg376 that appeared at the fourth position of the EXXR motif could be a key factor that mediates the interaction between the EXXR motif and the meander loop by hydrogen bonding with Glu373, Arg430, His413, Ala423, and Glu425 (Fig 4B). Zhao et al. [40] stated that Arg376 also has a hydrophobic interaction with the side chain of Trp419 (Fig 4B). In ′CWS5095′, the amino acid change Arg376Lys leads to multiple rotamers, which significantly reduces the number of hydrogen bonds (Fig 4C). In the mutant, the EXXR motif may not stably interact with the meander loop, which means that there is structural perturbation in CYP450 and inhibited heme binding. In summary, the noteworthy amino acid change (Arg376Lys) may lead to loss-of-function of Sg-5 protein. Therefore, there is no biosynthesis of group A saponins in ′CWS5095′. The mutant allele of ′CWS5095′ was designated as *sg-5a*.

### Co-segregation of the group A saponin phenotype with the *G*. *soja* mutant

**Fig 5:**
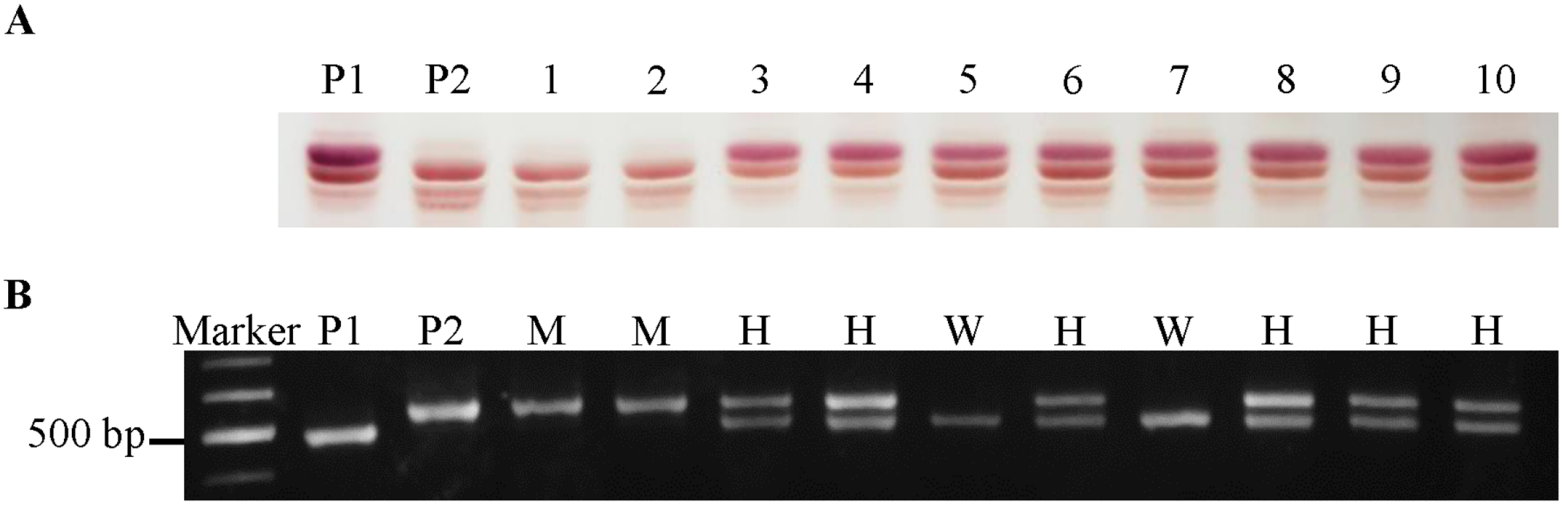
Co-segregation analysis of the *Sg-5* locus that controls group A saponin mutants. (A) Thin layer chromatogram showing the segregation of saponin phenotypes in the F_2_ populations derived from a cross between ′Uram′ and ′CWS5095′ (B) A dCAPS analysis of the *sg-5a* allele showing the co-segregation of *Sg-5* polymorphism and the saponin pattern detected in the F_2_ populations from a cross between ′Uram′ and ′CWS5095′. P1, ′Uram′; P2, ′CWS5095′; W, wild homozygote; H, heterozygote; M, mutant homozygote.

A derived cleaved amplified polymorphic sequence (dCAPS) analysis was performed to determine the co-segregation pattern of the *sg-5a* allele and the group A saponin phenotype (Fig 5). A pair of dCAPS primers amplified 525-bp DNA products. The products from the wild-type control soybeans (′Pungsannamul′ and ′Uram′) were digested with *Mbo*I to generate 476-bp DNA fragments, whereas the products from *sg-5a* (′CWS5095′) remained uncut (Fig 5B). F_2_ individuals that did not produce group A saponins only contained the longer fragment, whereas F_2_ individuals that did produce group A saponins either had only the shorter fragment or both the longer and shorter fragments (Fig 5B). These results indicate that the dCAPS marker co-segregated with the saponin phenotype of 120 F_2_ plants derived from a cross between ′Uram′ and ′CWS5095′ (*sg-5a*). Similarly, the saponin phenotype co-segregated with a dCAPS marker in 304 F_3_ progeny populations (from F_2_ heterozygous individual) derived from a cross between ′Pungsannamul′ and ′CWS5095′. The segregation fits a 1:2:1 ratio (Table 1). These results revealed a perfect co-segregation of the *Sg-5* polymorphism with the saponin phenotype, which indicated that the *Sg-5* locus is tightly linked to group A saponin biosynthesis in ′CWS5095′ and that the novel *sg-5a* allele is recessive to *Sg-5*.

## Discussion

Triterpenoid saponins are important secondary metabolites in soybean seeds. OSCs, CYP450s, and UGTs are the three main enzymes involved in the biosynthesis of saponins [3]. CYP450s are mono-oxygenases that contain heme as a cofactor. In flowering plant genomes, around 300 CYP450s genes have been identified [41]. Plant CYP450s were generally classified into two major clades: A-type and non-A-type clades [42]. Majority of A-type and a few non-A-type CYP450s are involved in the biosynthesis of secondary metabolites such as terpenoids, flavonoids, alkaloids and phytoalexins [42, 43]. One of the non-A-type CYP450s, CYP72, were found to participate in pentacyclic triterpene modifications [43,44]. The CYP72 family consists of three subfamilies, CYP72A, CYP72B and CYP72C [45]. The members of the CYP72A subfamily were found to be involved in the legume-specific triterpene saponin biosynthesis [46]. To date, four CYP72As of *Medicago truncatula* (CYP72A61, CYP72A63, CYP72A67, and CYP72A68) and one CYP72A of *Glycyrrhiza uralensis* (CYP72A154) were identified to be associated in the triterpene saponin biosynthesis [32,46,47]. In soybean, CYP93E1 is first identified as an A-type CYP450 associated with the triterpenoid saponin biosynthesis, which catalyzes the hydroxylation of β-amyrin at C-24 [48]. Recently, CYP72A69, which catalyzes the hydroxylation of C-21 during the biosynthesis of soyasapogenol A, was characterized (*Glyma*.*15g243300*) [17]. In the present study, we obtained the new mutant accession, ′CWS5095′ from the Chung wild legume germplasm collection [33,49]. Phenotypic analyses using TLC and LC-PDA/MS/MS showed that the mutant ′CWS5095′ did not produce any group A saponins. Segregation analysis showed that the absence of group A saponins is controlled by a single recessive allele. The locus that controls group A saponin biosynthesis in soybean was mapped to 4.3 Mb between the Affx-89193969 and Affx-89134397 SNP arrays on chromosome 15 (E) where the previously identified gene *Glyma*.*15g243300* is positioned.

The CYP450s were identified by primary structure analysis of the protein sequence, particularly the two CYP450 signature motifs. They are the FXXGXRXCXG (CXG) motif in the heme-binding domain and the EXXR motif in the K-helix [50]. Although all CYP450s have a similar structural fold, they commonly have less than 20% sequence identities [51]. There are only three amino acid residues that are completely conserved (Cys in the CXG motif, and Glu and Arg in the EXXR motif) in the CYP450 superfamily proteins across almost all the biological kingdom species [34,52]. The amino acids “E” and “R” in the EXXR motif are occasionally reported as being non-conserved [35,36]. The EXXR motif is believed to involved in stabilizing the core that is associated with the heme prosthetic group of CYP450s [37]. Mutations in either the glutamic acid or the arginine residues of the EXXR motif have led to impaired enzymatic actions in several CYP450s [53–55]. Analysis of the coding sequence for *Glyma*.*15g243300* in ′CWS5095′ revealed the presence of four SNPs compared to the wild-type control soybeans, ′Williams 82′ and ′Pungsannamul′. Among them, a significant amino acid change (R376K) was detected in the EXXR motif. The predicted 3D model for Sg-5 showed that the EXXR motif mediated the meander loop and heme. Based on the importance of the meander loop, which has a role in heme binding, EXXR should stably interact with the residues in the meander loop [37,55]. Furthermore, the Arg376 residue showed extensive interactions with several residues in the meander loop. Based on this structural feature, R376K in the mutant ′CWS5095′ may lead to structural perturbation and thereby decrease binding affinity for the heme cofactor. Higashimoto et al. [56] stated that replacement of Arg with Lys in the heme oxygenase-1 caused a loss of the heme degradation activity. This suggests that even the Arg and Lys have similar chemical features, the substitution of the Arg to Lys in the EXXR motif of CYP450 that is absolutely conserved in the plant kingdom may lead to a loss-of-function and consequently no biosynthesis of group A saponins in ′CWS5095′. In addition, our results showed a perfect co-segregation of the missense mutation (R376K) in the *sg-5a* allele with the absence of group A saponins in ′CWS5095′. However, we cannot rule out the possibility that other polymorphisms may also play a subtle role in the functional activity of CYP72A69. Additionally, analysis of the enzymatic activity is necessary to confirm the association of the R376K polymorphism with absence of group A saponins.

During the past three decades, several studies for genetic improvement have been conducted to eliminate undesirable components and enhance the quality of soybean seeds [57]. Soybean seeds contain undesirable components like raffinose, stachyose, lipoxygenase, and Kunitz trypsin inhibitor. Raffinose and stachyose accumulation in soybean seeds are indigestible and cause flatulence in poultry and livestock [58]. Qiu et al. [59] reported that a mutation in the stachyose synthase gene led to an extremely low content of stachyose. The undesirable ‘beany’ flavor in soybean seeds is caused by oxidation of soy products resulting from seed lipoxygenase activities [60]. Mutations in the *lipoxygenase-2* gene led to a reduction in the lipoxygenase activity and thereby a better-quality soybean meal [60–62]. Kunitz trypsin inhibitors (KTi) in soybean seeds also contribute to indigestibility [63], and mutations in the *KTi* gene results in a drastic reduction in the level of Kunitz trypsin inhibitors during the seed development stage [64].

In addition to the above mentioned undesirable traits, group A saponins in soybean seeds are well-known for unpleasant taste and bitterness in soybean food [65]. To the contrary, group B and DDMP saponins are less bitter and thus more beneficial to human health [16,24]. If group A saponins are removed genetically from soybean, group B saponins are increased in compensation for the elimination of group A saponins [8]. Therefore, the elimination of group A saponins and enrichment of group B and DDMP saponins in soybean seeds are in limelight in researches to improve flavor and enhance health benefits [8]. For this purpose, we are developing an elite cultivar containing no group A saponins by a series of backcrosses to eliminate the wild genetic background of *G*. *soja*. Later, the newly developed elite line will be used to prove the hypothesis that the absence of group A saponins lead to an increase of DDMP and group B saponins. Inheritance of the *sg-5a* mutant allele completely corresponding to the absence of group A saponins was detected by the SNP marker that we developed in this study. Therefore, the SNP marker would serve as a useful tool for high-throughput marker-assisted selection to develop a soybean cultivar with no group A saponins.

## Materials and methods

### Plant materials

A new *G*. *soja* mutant, ′CWS5095′, that does not contain any group A saponins, was discovered in Gyeonggi-do, Republic of Korea [33,49]. To isolate and identify the locus involved in group A saponin mutants, physical mapping was performed using segregating populations derived from crosses of the ′Pungsannamul′ and ′Uram′ cultivars with ′CWS5095′. In addition, three wild *G*. *soja* accessions (′CW12048′, ′CW16078′, and ′CW13613′) were also used as wild-type controls. All the experimental populations were grown in the experimental fields at Kyungpook National University (Gunwi, 36°07′N, 128°38′E, Republic of Korea).

### Saponin extraction and thin layer chromatography analysis of ′CWS5095′

Saponins were extracted from the hypocotyls of mature dry seeds from each accession used in this study. Saponin extractions were kept for 24 h in ten-fold volumes of (v/w) of 80% (v/v) methanol (aqueous) at room temperature. The extracts were directly used for TLC analysis or stored at 4°C. The TLC was performed according to Krishnamurthy et al. [66], with slight modifications. Briefly, 5 μL of saponin extracts from each sample were directly loaded on pre-silica gel plates (TLC silica gel 60 F_254_, Merck, Darmstadt, Germany) with a micropipette and air dried. The dried plates were developed in a developing chamber, which was saturated with the lower phase of a chloroform:methanol:water (65:35:10, v/v/v) mix for 50 min. The plates were dried at 90°C for 5 min and then developed by freshly prepared H_2_SO_4_ for 8 min in a closed chamber. The saponins were visualized by heating at 100–110°C for 10 min.

### LC-PDA/MS/MS analysis

To obtain the detailed composition of the saponins in ′CWS5095′, a LC-PDA/MS/MS on a C30 reverse phase column was performed according to Takada et al. [8]. The saponins were extracted from the hypocotyls and cotyledons of each accession by adding 10 and 20 μL of 50-fold and 7.5-fold (v/w) solutions of 80% aqueous methanol (v/v), respectively. The UV and MS spectra were analyzed using Xcalibur software version 3.1 (Thermo Fisher, Santa Clara, CA, USA).

### Physical mapping of the *Sg-5* locus and sequence analysis of the *Sg-5* gene

Genomic DNAs were isolated from trifoliate leaves using the CTAB extraction method [67]. To identify the locus involved in group A saponin mutants, a physical map was constructed using an Affymetrix 180K Axiom^®^ SNP array from the crosses between ′Pungsannamul′ and ′CWS5095′, and between ′Uram′ and ′CWS5095′. A total of 20 F_2_ individuals were selected after phenotyping of F_3_ populations (mutant-type, nine; wild-type, five; heterozygote, six) for physical map construction. The *Sg-5* locus was demarked by the detection of recombinants in the F_2_ individuals through Microsoft Excel software. The coding sequences of the candidate *Sg-5* gene (*Glyma*.*15g243300*) were amplified using the following PCR conditions: initial denaturation at 94°C for 5 min, 35 cycles of denaturation at 94°C for 20 s, annealing at 58°C for 40 s, extension at 72°C for 1 min; and a final extension at 72°C for 5 min. The PCR products were subjected to sequencing (SolGent Co., Daejeon, Republic of Korea). The primers used for the sequencing are listed in Table 2.

**Table 2:** List of primers.

Analysis	Primer name	Sequence (5′-3′)
*Sg-5*	Sg-5-F1	CACGCTTTTTGCATTTATCC
	Sg-5-R1	CGAACAAAACTTGGTTCCTTG
	Sg-5-F2	AGCTCGCAAAGTTGCTCATT
	Sg-5-R2	GCTGATGTTGCTTGCAGTTG
	Sg-5-F3	CAACTGCAAGCAACATCAGC
	Sg-5-R3	GAAGGGAATGTTCTTTGATGC
	Sg-5-F4	GTGATCAAACTTATTGGATGAGC
	Sg-5-R4	TCCCGTGTTCTTCAATTTCC
	Sg-5-F5	GGTTGGTACCTAAAAGGATGAA
	Sg-5-R5	TTCATATTTCTCCACCTTATG
	Sg-5-F6	ATCATTTCCTGCTGGAGTGG
	Sg-5-R6	ATGGTGACATTCTAACTCCACAA
dCAPS	sg-5-dCAPS-F	GGTTGGTACCTAAAAGGATGAA
	sg-5-dCAPS-R	ACATCTTTGATAACTTTTCGAGGAACACCAACTCCTGGAGGGTATGAT

### Multiple alignment analysis and 3D structure modeling of the Sg-5 protein

Multiple alignment analysis was performed using the ClustalW program (http://www.genome.jp/tools-bin/clustalw). CYP450 superfamily proteins derived from the NCBI-CDD (https://www.ncbi.nlm.nih.gov/Structure/cdd/cdd.shtml) were used to construct a phylogenetic tree and for multiple alignment analysis, which demonstrated the influence of the unique SNPs detected in ′CWS5095′. A 3D model of the Sg-5 protein was developed using Phyre2, a web-based 3D structure modeling program [38].

### Derived cleaved amplified polymorphic sequence analysis

Genomic DNAs were isolated from all F_2_ individuals derived from crosses between ′Pungsannamul′ ′CWS5095′, and between ′Uram′ and ′CWS5095′. They were used for dCAPS analysis. The PCR primers (Table 2) were designed to detect a single-base substitution at nucleotide position 1127 of *Sg-5* in ′CWS5095′. The nucleotide substitution (G to A) generates a *Mbo*I site (GATC) in the amplified PCR product from the wild parents. The PCR conditions were the same as those mentioned above. The amplified products were digested with *Mbo*I (Takara Bio Inc, Shiga, Japan) and separated on a 1.2% agarose gel.

## Supporting information

S1 Fig Chemical structure and nomenclatures of triterpene saponins in soybean. (A) Representative chemical structures of group A, DDMP, group B and group E saponins. (B) Classification of soybean saponins based on their sugar moieties or the DDMP moiety attached to the C-3 and C-22 positions. Asterisks indicate the saponins analyzed in this study.
(PDF)

S2 Fig Separation and detection of saponin components from soybean seed cotyledons. Seed cotyledon extracts (80% aqueous methanol) of ′Pungsannamul′ (wild-type) and ′CWS5095′ (mutant) were separated by high-performance liquid chromatography (HPLC) and detected by UV absorption at 205 nm. Chemical structures of saponins corresponding to each peak were identified using LC-PDA/MS/MS analysis. Chemical structures and the corresponding names of the saponins are shown in Fig 1 and S1 Fig.
(PDF)

S3 Fig Physical map construction of the *Sg-5* locus in soybean. (A) Mapping of the *Sg-5* locus on chromosome 15 (linkage group E) with individual F_2_ lines from populations derived from crosses between ′Pungsannamul′ and ′CWS5095′ and between ′Uram′ and ′CWS5095′. The previously mapped *Sg-5* locus along with the SSR markers (Satt117 and GMES0332) by Takada et al. (2013) was indicated. (B) Phenotype and genotype of the recombinants. The saponin phenotype of each recombinant was determined by progeny testing. Horizontal bars represent the recombinant region for each F_2_ individual. Black, white and shaded bars represent, respectively, mutant homozygous, wild homozygous and wild heterozygous F_2_ individuals.
(PDF)

S4 Fig Comparison of amino acid sequence Sg-5 proteins from ′CWS5095′, two wild soybean accessions, and 18 CYP450 superfamily proteins belonging to other plant species. GenBank accession numbers and sources of CYP450 superfamily proteins are listed below. The single nucleotide changes detected in ′CWS5095′ are highlighted by yellow and black boxes. The EXXR motif is shown by a red box. The single nucleotide polymorphisms detected in ′CW13613′ are highlighted in blue.
(PDF)
