# Supplementary material for: Molecular elucidation of a new allelic variation at the Sg-5 gene associated with the absence of group A saponins in wild soybean
Source: PLoS One. 2018 Jan 30;13(1):e0192150. doi: 10.1371/journal.pone.0192150 (PMC5790262; doi:10.1371/journal.pone.0192150)
Supplement: S1 Fig — (A) Representative chemical structures of group A, DDMP, group B and group E saponins. (B) Classification of soybean saponins based on their sugar moieties or the DDMP moiety attached to the C-3 and C-22 positions. Asterisks indicate the saponins analyzed in this study. (PDF) [file pone.0192150.s001.pdf]

A

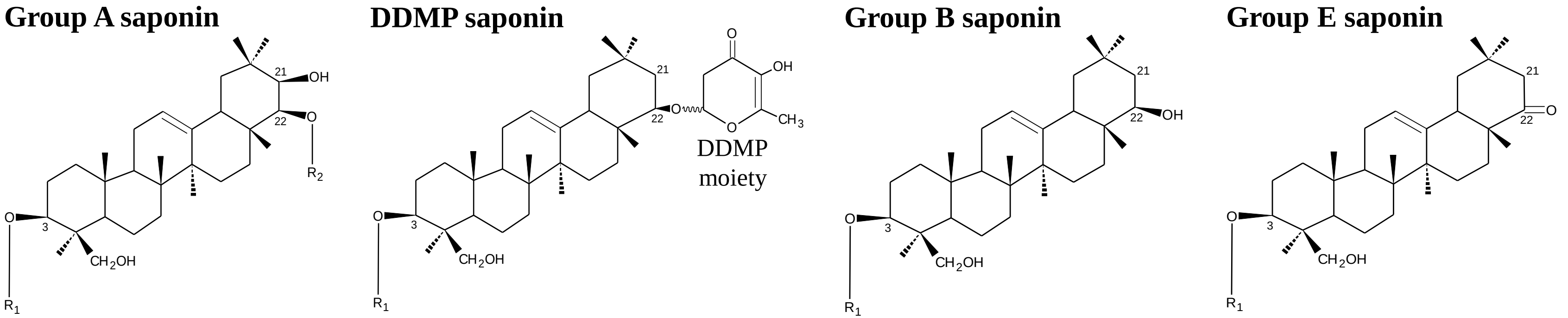

B

R1 (C-3 position)	C-22 position					
	Soyasapogenol-A (R_2)			Soyasapogenol-B		
	Group A saponin			DDMP	Group B	Group E
	—Ara-acetylXyl	—Ara-acetylGlc	—Ara	—DDMP	—HO	=O
—GlcUA-Gal-Glc	Aa	Ab*	A0-αg	αg	Ba*	Bd*
—GlcUA-Gal-Rha	Au	Ac	A0-βg	βg*	Bb*	Be
—GlcUA-Gal	Ae	Af*	A0-γg	γg*	Bb′	Be′
—GlcUA-Ara-Glc	Ax	Ad	A0-αa	αa	Bx	Bf
—GlcUA-Ara-Rha	Ay	Az	A0-βa	βa*	Bc*	Bg
—GlcUA-Ara	Ag	Ah*	A0-γa	γa*	Bc′	Bg′*
