# Supplementary figures and images for: Molecular elucidation of a new allelic variation at the Sg-5 gene associated with the absence of group A saponins in wild soybean
Source: PLoS One. 2018 Jan 30;13(1):e0192150. doi: 10.1371/journal.pone.0192150 (PMC5790262; doi:10.1371/journal.pone.0192150)

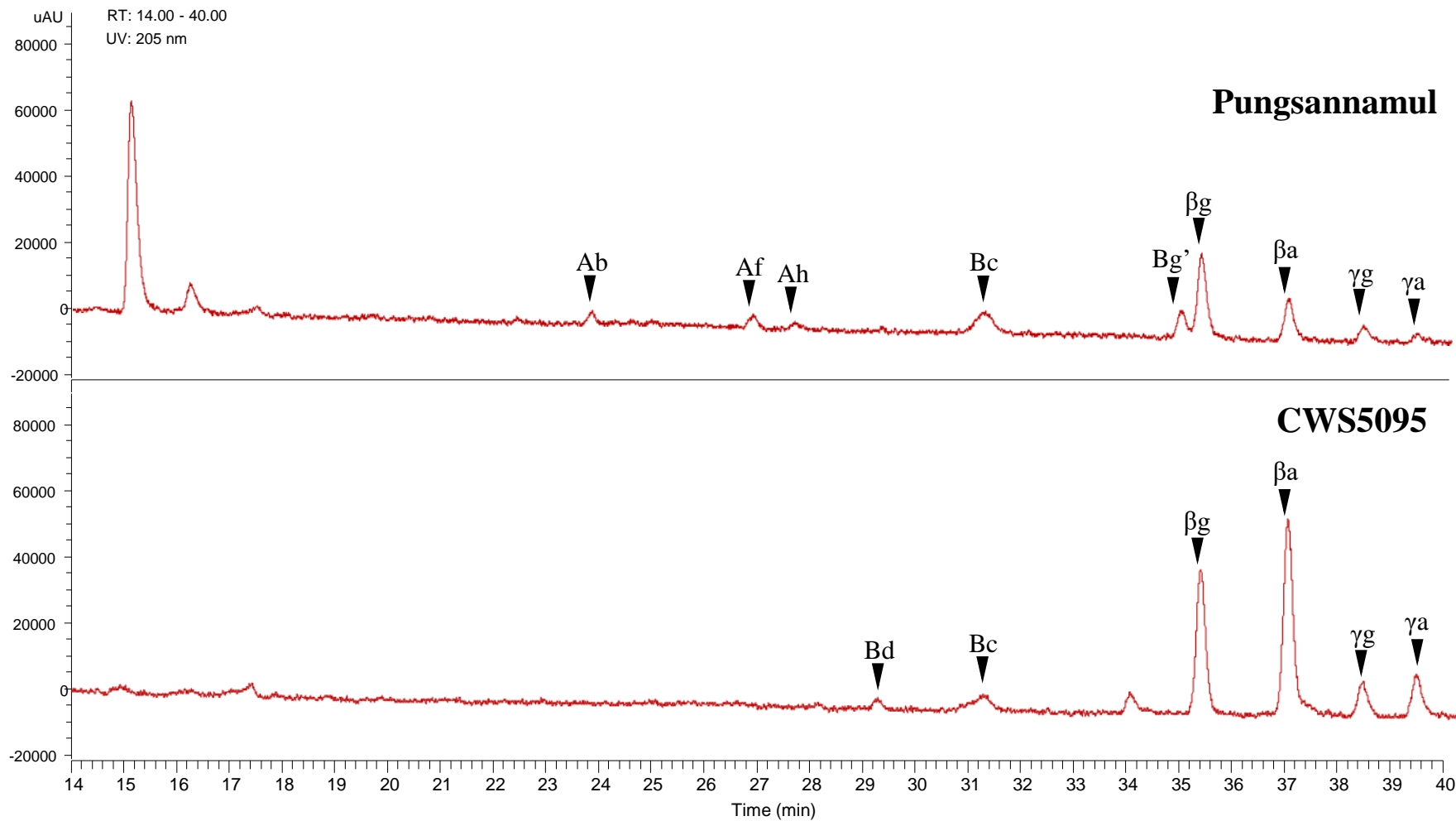

Supplement: S2 Fig — Seed cotyledon extracts (80% aqueous methanol) of ′Pungsannamul′ (wild-type) and ′CWS5095′ (mutant) were separated by high-performance liquid chromatography (HPLC) and detected by UV absorption at 205 nm. Chemical structures of saponins corresponding to each peak were identified using LC-PDA/MS/MS analysis. Chemical structures and the corresponding names of the saponins are shown in Fig 1 and S1 Fig. (PDF) [file pone.0192150.s002.pdf]

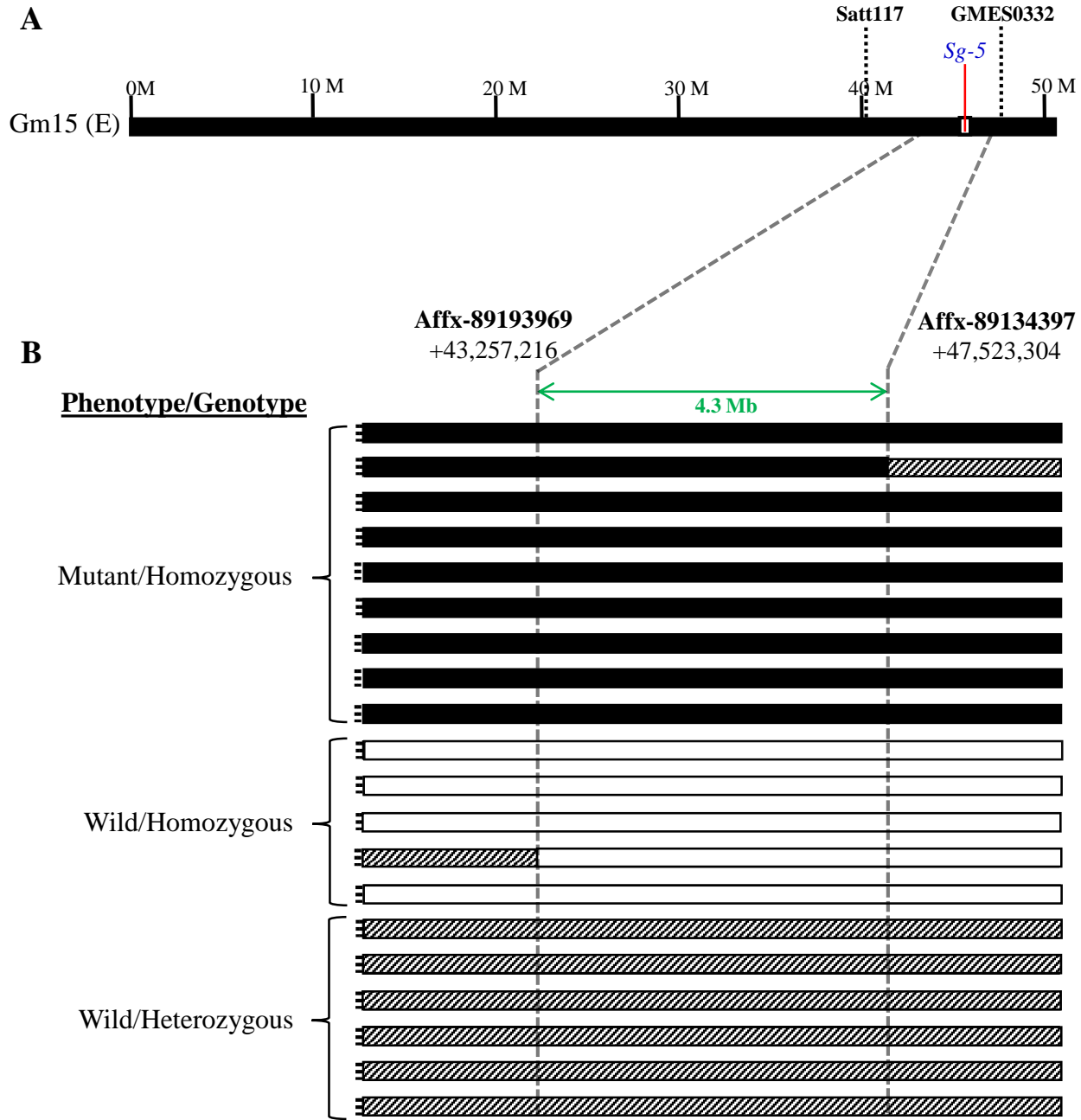

Supplement: S3 Fig — (A) Mapping of the Sg-5 locus on chromosome 15 (linkage group E) with individual F2 lines from populations derived from crosses between ′Pungsannamul′ and ′CWS5095′ and between ′Uram′ and ′CWS5095′. The previously mapped Sg-5 locus along with the SSR markers (Satt117 and GMES0332) by Takada et al. (2013) was indicated. (B) Phenotype and genotype of the recombinants. The saponin phenotype of each recombinant was determined by progeny testing. Horizontal bars represent the recombinant region for each F2 individual. Black, white and shaded bars represent, respectively, mutant homozygous, wild homozygous and wild heterozygous F2 individuals. (PDF) [file pone.0192150.s003.pdf]
