# Supplementary material for: Molecular elucidation of a new allelic variation at the Sg-5 gene associated with the absence of group A saponins in wild soybean
Source: PLoS One. 2018 Jan 30;13(1):e0192150. doi: 10.1371/journal.pone.0192150 (PMC5790262; doi:10.1371/journal.pone.0192150)
Supplement: S4 Fig — GenBank accession numbers and sources of CYP450 superfamily proteins are listed below. The single nucleotide changes detected in ′CWS5095′ are highlighted by yellow and black boxes. The EXXR motif is shown by a red box. The single nucleotide polymorphisms detected in ′CW13613′ are highlighted in blue. (PDF) [file pone.0192150.s004.pdf]

S4 Fig.

```
         10        20        30        40        50        60        70        80        90       100
....|....|....|....|....|....|....|....|....|....|....|....|....|....|....|....|....|....|....|....|
-------------------MEAAWVNILMLILILALIWVWKKFNSLWLTPKRLEKI---------LREQGLRGSPYRFKVGDTKETLKMQMQAMSKPMNL Glycine max
-------------------MEAAWDNILMLILILALIWVWKKFNSLWLTPKRLEKI---------LREQGLRGSPYRFKVGDTKETLKMQMQAMSKPMNL CWS5095 (G. soja)
-------------------MEAAWVNILMLILILALIWVWKKFNSLWLTPKRLEKI---------LREQGLRGSPYRFKVGDTKETLKMQMQAMSKPMNL CW13613 (G. soja)
-------------------MEAAWVNILMLILILALIWVWKKFNSLWLTPKRLEKI---------LREQGLRGSPYRFKVGDTKETLKMQMQAMSKPMNL CW16078 (G. soja)
-----------------MMVTLVLKYVLVIVMTLILRVLYDSICCYFLTPRRIKKF---------MERQGITGPKPRLLTGNIIDISKMLSHSASNDCSS PLN02290
---------------------MDSPLLQSLQSPSTLFILASLFVSLILWFLIRKKL---------PYPPGPKGYP---IIGNLGMVDQLT---------- PLN02183
MDTLASNSSDLTTKSSLGMSSFTNMYLLTTLQALAALCFLMILNKIKSSSRNKKLH---------PLPPGPTGFP---IVGMIPAMLKNRPV-------- PLN02971
----------------------MAFTAFLLLLS--SIAAGFLLLLRRTRYRRMG------------LPPGSLGLP---LIGETFQLIGAYKTE------- PLN02987
------------------MVLLWELTMAALFFFINYLLTRCLIRKLSTR----------------QLPPGPRGWP---IIGAIPVLGAMP---------- PLN00110
--------------------------MAMLGFYVTFIFFLVCLFTYFFLQKKPQGQ------------PILKNWP---FLRMLPGMLHQIPR-------- PLN02169
-------------------MDISALFLTLFAGSLFLYFLRCLISQRRFGSSKLP------------LPPGTMGWP---YVGETFQLYSQDPN-------- PLN02196
----------------------MVLVAIGVVVAAAVVVSSLLLRWNEVRYSRKRG-----------LPPGTMGWP---LFGETTEFLKQGPS-------- PLN02774
---------------------MEDIIIGVVA-------LAAVLLFFLYQKPKTKRY---------KLPPGPSPLP---VIGNLLQLQKLNP--------- PLN02966
---MMSFTTSLP---------YPFHILLVFILSMASITLLGRILSRPTKTKDR-SC---------QLPPGPPGWP---ILGNLPELFMTRPR-------- PLN03018
----------------------MDLLLIIAG-------LVAAAAFFFLRSTTKKSL---------RLPPGPKGLP---IIGNLHQMEKFNP--------- PLN03234
---------------------MELTWLLLLATLLLSTTLLVFLFHGGSSATGGEKRR--------RLPPGPATVP---VLGNLLWATNSGMD-------- PLN00168
----------------MESGSIWVVLAVIFGGLGVGKWILKKVNWWLYEAQLGEKQ--------YSLPPGDLGWP---FIGNMWSFLRAFKSS------- PLN02302
-MAASVVRVAIATGASLAVHLFVKSFLQAQHPALTLLLPVAVFAGIAVGAKGGNGGD-------GKAPPGPAAVP---VFGNWLHVGNDLN--------- PLN02394
---------------------MDATTTVSMEMELPWGARCAGLAFFAFSVCLAALG---------VVLLVARRWPWCSCHVCRAYLTGSWAR-------- PLN02426
----------------MFETEHHTLLPLLLLPSLLSLLLFLILLKRRNRKTRFN------------LPPGKSGWP---FLGETIGYLKPYTAT------- PLN02500
-----------------MEVAAMEISTSLLLTTVALSVIVCYALVFSRAGKARAPL---------PLPPGPRGWP---VLGNLPQLGGKT---------- PLN02687
----------------MDTSSSLLFFSFFFFIIIVIFNKINGLRSSPASKKKLNDHHVTSQSHGPKFPHGSLGWP---VIGETIEFVSSAYSD------- PLN03141

        110       120       130       140       150       160       170       180       190       200
....|....|....|....|....|....|....|....|....|....|....|....|....|....|....|....|....|....|....|....|
FSNDIGPRVSPYDHYIVNKHGK--NSFIWNGQTPRVTLTDPELIKDVFN-KIYDFGKPNMGPN--IRSLIP-GLAMHE-GEKWSKHRKIINP-AFNLEKL Glycine max
FSNDIGPRVSPYDHYIVNKHGK--NSFIWNGQTPRVTLTDPELIKDVFN-KIYDFGKPNMGPN--IRSLIP-GLAMHE-GEKWSKHRKIINP-AFNLEKL CWS5095 (G. soja)
FSNDIGPRVSPYDHYIVNKHGK--NSFIWNGQTPRVTLTDPELIKDVFN-KIYDFGKPNMGPN--IRSLIP-GLAMHE-GEKWSKHRKIINP-AFNLEKL CW13613 (G. soja)
FSNDIGPRVSPYDHYIVNKHGK--NSFIWNGQTPRVTLTDPELIKDVFN-KIYDFGKPNMGPN--IRSLIP-GLAMHE-GEKWSKHRKIINP-AFNLEKL CW16078 (G. soja)
IHHNIVPRLLPHYVSWSKQYGK--RFIMWNGTEPRLCLTETEMIKELLT-KHNPVTGKSWLQQQGTKGFIGRGLLMAN-GEAWHHQRHMAAP-AFTRDRL PLN02290
---------HRGLASLSKRYGG--LCHLQMGGLHVVAVSTPEIAREVLQAQDVVFANRPANVAIVYLTYDRADMAFANYGPFWRQTRKICVMKLFSRKRA PLN02183
--------FRWLHSLMKELNTE--IACVRLGNTHVIPVTCPKIAREIFKQQDALFASRPLTYAQKILSNGYKTCVITPFGEQFKKMRKVIMTEIVCPARH PLN02971
-------NPEPFIDERVARYGS--VFMTHLFGEPTIFSADPETNRFVLQNEGKLFECSYPASICNLLGKHS---LLLMKGSLHKRMHSLTMSFANSSIIK PLN02987
---------HAALAKMAKQYGP--VMYLKMGTCNMVVASTPDAARAFLKTLDLNFSNRPPNAGATHLAYNAQDMVFADYGPRWKLLRKLSNLHMLGGKAL PLN00110
-------IYDWTVEVLEATNLTFYFKGPWLSGTDMLFTADPRNIHHILSSNFGNYPKGPEFKKIFDVLGEG---ILTVDFELWEEMRKSNHALFHNQDFI PLN02169
----------VFFQSKQKRYGS--VFKTHVLGCPCVMISSPEAAKFVLVTKSHLFKPTFPASKERMLGKQA---IFFHQGDYHAKLRKLVLRAFMPESIR PLN02196
-----------FMKARRLRYGS--VFRTHILGCPTVVCMEAELNRRALASEGRGFVPGYPQSMLDILGRNN---IAAVQGPLHRAMRGAMLSLVRPAMIR PLN02774
---------QRFFAGWAKKYGP--ILSYRIGSRTMVVISSAELAKELLKTQDVNFADRPPHRGHEFISYGRRDMALNHYTPYYREIRKMGMNHLFSPTRV PLN02966
--------SKYFRLAMKELKTD--IACFNFAGIRAITINSDEIAREAFRERDADLADRPQLFIMETIGDNYKSMGISPYGEQFMKMKRVITTEIMSVKTL PLN03018
---------QHFLFRLSKLYGP--IFTMKIGGRRLAVISSAELAKELLKTQDLNFTARPLLKGQQTMSYQGRELGFGQYTAYYREMRKMCMVNLFSPNRV PLN03234
--------IMRAVRRLHARHGP--MLGLRMGSRLEVIVADRRLAHAALVESGAAMADRPEFASRALLGLDTATISNSSYGPLWRLFRRNFVAEVAHPARL PLN00168
-------DPDSFMRTFINKYGDNGIYKAFMFGNPSVFVTTPEACRRVLS-NDDAFKPGWPISTLKLIGRKS---FIDISYEEHKRLRRLTSAPVNGHEAL PLN02302
---------HRFLAAMSARYGP--VFRLRLGVRNLVVVSDPKLATEVLHTQGVEFGSRPRNVVFDIFTANGADMVFTEYGDHWRRMRRVMTLPFFTARVV PLN02394
-------EFTNLGDWYAHLLRRSPTGTVHVHVLGCTVTANPANVEHMLRTRFDNFPKGRPFAALLGDLLGDG--IFNVDGHAWRHQRKMASLELGSVAVR PLN02426
-------TLGDFMQQHVSKYGK--IYRSNLFGEPTIVSADAGLNRFILQNEGRLFECSYPRSIGGILGKWS---MLVLVGDMHRDMRSISLNFLSHARLR PLN02500
---------HQTLHEMTKVYGP--LIRLRFGSSDVVVAGSAPVAAQFLRTHDANFSSRPRNSGGEHMAYNGRDVVFGPYGPRWRAMRKICAVNLFSARAL PLN02687
-------RPESFMDKRRLMYGR--VFKSHIFGTATIVSTDAEVNRAVLQSDSTAFVPFYPKTVRELMGKSS---ILLINGSLHRRFHGLVGSFLKSPLLK PLN03141

        210       220       230       240       250       260       270       280       290       300
....|....|....|....|....|....|....|....|....|....|....|....|....|....|....|....|....|....|....|....|
KNMLPLFIQCCDDLISKWEEMLSSDGS---SEIDVWPFVKNLTADVISRTAFGSSYLEGRR--------------IFQLLKEKIELTLKMR--------G Glycine max
KNMLPLFIQCCDDLISKWEEMLSSDGS---SEIDVWPFVKNLTADVISRTAFGSSYLEGRR--------------IFQLLKEKIELTLKMR--------G CWS5095 (G. soja)
KNMLPLFIQCCDDLISKWEEMLSSDGS---SEIDVWPFVKNLTADVISRTAFGSSYLEGRR--------------IFQLLKEKTELILKMR--------G CW13613 (G. soja)
KNMLPLFIQCCDDLISKWEEMLSSDGS---SEIDVWPFVKNLTADVISRTAFGSSYLEGRR--------------IFQLLKEKIELTLKMR--------G CW16078 (G. soja)
KGYAKHMVECTKMMAERLRKEVG-------EEVEIGEEMRRLTADIISRTEFGSSCDKGKE--------------LFSLLTVLQRLCAQATRHLCFP--G PLN02290
ESWASVR-DEVEFTVRRVSEKTG-------EPVNIGELVFALTRSITYKAAFGSS--SNE-----------GQEEFMEILQEFSKLFGAFNVADFFPWLG PLN02183
RWLHDNRAEETDHLTAWLYNMVKNS-----EPVDLRFVTRHYCGNAIKRLMFGTRTFSEK--TEADGGPTLEDIEHMDAMFEGLGFTFAFCISDYLP-ML PLN02971
DHLMLDIDRLVRFNLDSWS-----------SRVLLMEEAKKITFELTVKQLMSFDPGE----------------WSESLRKEYLLVIEGFFS-------L PLN02987
EDWAHVRVSELGHMLRAMCEASRKG-----EPVVVPEMLTYAMANMIGQIILSRRVFVTKG--------SE-SNEFKDMVVELMTSAGLFNVGDYIPSVA PLN00110
ELSVSSNKSKLKEGLVPFLDNAAQKN----IIIELQDVFQRFMFDTSSILMTGYDPMSLSIEML--------EVEFGEAADIGEEAIYYRHFKPVILWRL PLN02169
N-MVPDIESIAQDSLRSWEGT----------MINTYQEMKTYTFNVALLSIFGKDEVL----------------YREDLKRCYYILEKGYNS-------M PLN02196
SSLLPKIDAFMRSHLAAWSSSSSS------AVVDIQAKTKEMALLSALRQIAGVSAGP----------------LSDALKAELYTLVLGTIS-------L PLN02774
ATFKHVREEEARRMMDKINKAADKS-----EVVDISELMLTFTNSVVCRQAFGKK--YNED--------GEEMKRFIKILYGTQSVLGKIFFSDFFPYCG PLN02966
KMLEAARTIEADNLIAYVHSMYQRS-----ETVDVRELSRVYGYAVTMRMLFGRRHVTKENVFSDDGRLGNAEKHHLEVIFNTLNCLPSFSPADYVERWL PLN03018
ASFRPVREEECQRMMDKIYKAADQS-----GTVDLSELLLSFTNCVVCRQAFGKR--YNEY--------GTEMKRFIDILYETQALLGTLFFSDLFPYFG PLN03234
RQFAPARAAVLEELTDKLRRRQEDAG-----AGTILETFQYAMFFLLVAMCFGELLDERAVR------------DIAAAQRDLLLHSSKKLRVFAFLPAI PLN00168
SVYIPYIEENVIAMLEKWTTMG---------KIEFLTQVRKLTFKIIMYIFLSSES----------------EVVMEALEKDYTTLNYGVRA-------M PLN02302
QQYKAMWEAEMDAVVDDVRGDAVAQG----TGFVVRRRLQLMLYNIMYRMMFDARFESVDDP---------MFIEATRFNSERSRLAQSFEYNYGDFIPI PLN02394
SYAYKIIAQEVEARLMPVLADAADRG----AVLDLQDVFRRFAFDNICKISFGLDPGCLDREMP-------VSELADAFDAASRLSAMRGAAASPLLWRA PLN02426
TILLKDVERHTLFVLDSWQQN---------SIFSAQDEAKKFTFNLMAKHIMSMDPGEE---------------ETEQLKKEYVTFMKGVVS-------A PLN02500
DDLRAFREREAVLMVRSLAEASAAPGSSSPAAVVLGKEVNVCTTNALSRAAVGRRVFAAGA--------GEGAREFKEIVLEVMEVGGVLNVGDFVPALR PLN02687
AQIVRDMHKFLSESMDLWSED---------QPVLLQDVSKTVAFKVLAKALISVEKGE----------------DLEELKREFENFISGLMS-------L PLN03141
```

```
       310       320       330       340       350       360       370       380       390       400
....|....|....|....|....|....|....|....|....|....|....|....|....|....|....|....|....|....|....|....|
QRLVP----KRMKEIDRDIKASLMDIINKRDKALKAG--------------EATKN--NLLDILLESNHKEIEEH----------GNNKNVGMNIEEVIE Glycine max
QRLVP----KRMKEIDRDIKASLMDIINKRDKALKAG--------------EATKN--NLLDILLESNHKEIEEQ----------GNNKNVGMNLEEVIE CWS5095 (G. soja)
QRLVP----KRMKEIDRDIKASLMDIINKRDKALKAG--------------EATKN--NLLDILLESNHKEIEEQ----------GNNKNVGMNLEEVIE CW13613 (G. soja)
QRLVP----KRMKEIDRDIKASLMDIINKRDKALKAG--------------EATKN--NLLDILLESNHKEIEEQ----------GNNKNVGMNLEEVIE CW16078 (G. soja)
SRFLPSKYNREIKSLKTEVERLLMEIIDSRKDSVEIG--------------RSSSYGDDLLGLLLN----QMDSN----------KNN----LNVQMIMD PLN02290
WVN-AQDFNKRLAKARNSLDGFIDTIIDEHIAKKNNTKSL-------NAKDENEEVDSDMVDELLAFYSEDASKND-------FDESRSTVKFNKDHIKA PLN02183
TGLDLNGHEKIMRESSAIMDKYHDPIIDERIKMWRE---------------GKRTQIEDFLDIFISIKDEAG-----------------QPLLTADEIKP PLN02971
PLPLFSTTYRKAIQARRKVAEALTVVVMKR--------------------REEEEEGAERKKDMLAALLAADDG------------------FSDEEIVD PLN02987
WMD-LQGIERGMKRLHRRFDVLLTKMMEEHIA---------------TAHERKGKP--DFLDVLMANQEN---------------LDGE--KLSFTNIKA PLN00110
QNWIGIGLERKMRTALATVNRMFAKIISSRRKEEISR-------------AKTEPYSKDALTYYMNVDTSKYKLLK---------------PNKDKFIRD PLN02169
PVNLPGTLFHKSMKARKELSQILARILSER--------------------RQNGSSHNDLLGSFMGDKEE----------------------LTDEQIAD PLN02196
PINLPGTNYYQGFKARKKLVAMLEQMIAER--------------------RSSGQVHDDMLDALLTGVEGTREK------------------LTDEQIID PLN02774
FLDDLSGLTAYMKECFERQDTYIQEVVNETLDPKR-------------VKPETE----SMIDLLMGIYKEQP----------------FASEFTVDNVKA PLN02966
RGWNVDGQEKRVTENCNIVRSYNNPIIDERVQLWREE--------------GGKAAVEDWLDTFITLKDQNG-----------------KYLVTPDEIKA PLN03018
FLDNLTGLSARLKKAFKELDTYLQELLDETLDPNR-------------PKQETE----SFIDLLMQIYKDQP----------------FSIKFTHENVKA PLN03234
TTRLFAGRMKAMIAMRQRLKGMFMPLIDARRARKNLVDDHGDATAPPPPAASATTLPHSYVDTLLNLRINDNGGE--------------RALTDDEMVA PLN00168
AINLPGFAYYKALKARKRLVAIFQSIVDERRNLRKNS--------------ARNAKKKDMMDSLLGVEDENGRK------------------LTDEEIID PLN02302
LRPFLRGYLNKCRDLQSRRLAFFNNNYVEKRRKVMDT-------------PGDRNKLRCAIDHILEAEKNGE--------------------LTAENVIY PLN02394
KRFLNVGSERELRKAIKVVDELAAAMIRERQK-------------------LGVGSSHDLLSRFMASTGVDDAAA------------------DDKFLRD PLN02426
PLNLPGTAYHKALQSRATILKFIERKMEERKLDI----------------KEEDQEEEEVKTEDEAEMSKSDHVRKQRTDDDLLGWVLKHSNLSTEQILD PLN02500
WLD-PQGVVARMKKLHRRFDDMMNAIIAERRAGSL---LK-------PTDSREEGK--DLLGLLLAMVQEQE-----------WLAAGEDDRITDTEIKA PLN02687
PINFPGTQLHRSLQAKKNMVKQVERIIEGKIR------------------KTKNKEEDDVIAKDVVDVLLKDSS----------------EHLTHNLIAN PLN03141

       410       420       430       440       450       460       470       480       490       500
....|....|....|....|....|....|....|....|....|....|....|....|....|....|....|....|....|....|....|....|
ECKLFYFAGQDTTSVLLVWTMILLSRYPDWQARAREEVSQVFGNQ------KPTFDGLNQLKIVTMILYEVLRLYPPG-VGVPRKVIKDVKLGNLSFPAG Glycine max
ECKLFYFAGQDTTSVLLVWTMILLSRYPDWQARAREEVSQVFGNQ------KPTFDGLNQLKIVTMILYEVLKLYPPG-VGVPRKVIKDVKLGNLSFPAG CWS5095 (G. soja)
ECKLFYFAGQDTTSVLLVWTMILLSRYPDWQARAREKVSQVFGNQ------KPTFDGLNQLKIVTMILYEVLRLYPPG-VGVPRKVIKDVKLGNLSFPAG CW13613 (G. soja)
ECKLFYFAGQDTTSVLLVWTMILLSRYPDWQARAREKVSQVFGNQ------KPTFDGLNQLKIVTMILYEVLRLYPPG-VGVPRKVIKDVKLGNLSFPAG CW16078 (G. soja)
ECKTFFFTGHETTSLLLTWTLMLLAHNPTWQDNVRDEVRQVCGQDG-----VPSVEQLSSLTSLNKVINESLRLYPPA-TLLPRMAFEDIKLGDLIIPKG PLN02290
LIMDVMFGGTETVASAIEWAIAELMKSPEDLKKVHQELMDVVGLNR-----TVHESDLEKLIYLKCAMKETLRLHPPIPLLLH-ETAKDTVLNGYRIPAR PLN02183
TIKELVMAAPDNPSNAVEWAIAEMINKPEILHKAMEEIDRVVGKER-----FVQESDIPKLNYVKAIIREAFRLHPVAAFNLPHVALSDTTVAGYHIPKG PLN02971
FLVALLVAGYETTSTIMTLAVKFLTETPLALAQLKEEHEKIRAMKSDS--YSLEWSDYKSMPFTQCVVNETLRVANIIGGVFR-RAMTDVEIKGYKIPKG PLN02987
LLLNLFTAGTDTSSSIIEWSLAEMLKNPRILKQAQDEMDQVIGRNR-----RLEESDIPKLPYLQAICKETFRKHPSTPLNLPRIADQACEVNGYYIPKG PLN00110
VIFSLVLAGRDTTSSVLTWFFWLLSKHPQVMAKLRHEINTK-----------FDNEDLEKLVYLHAALSESMRLYPPLPFNHKSPAKPDVLPSGHKVDAN PLN02169
NIIGVIFAARDTTASVMSWILKYLAENPNVLEAVTEEQMAIRKDKEEGE--SLTWGDTKKMPLTSRVIQETLRVASILSFTFR-EAVEDVEYEGYLIPKG PLN02196
LIITLIYSGYETMSTTSMMAVKYLSDHPKALEQLRKEHFDIRKGKAPED--AIDWNDFKSMTFTRAVIFETLRLATVVNGLLR-KTTQDVEMNGYVIPKG PLN02774
VILDIVVAGTDTAAAAVVWGMTYLMKYPQVLKKAQAEVREYMKEKGST---FVTEDDVKNLPYFRALVKETLRIEPVIPLLIPRACIQDTKIAGYDIPAG PLN02966
QCVEFCIAAIDNPANNMEWTLGEMLKNPEILRKALKELDEVVGRDR-----LVQESDIPNLNYLKACCRETFRIHPSAHYVPSHLARQDTTLGGYFIPKG PLN03018
MILDIVVPGTDTAAAVVVWAMTYLIKYPEAMKKAQDEVRSVIGDKG-----YVSEEDIPNLPYLKAVIKESLRLEPVIPILLHRETIADAKIGGYDIPAK PLN03234
LCSEFLNGGTDTTSTALEWIMAELVKNPTIQDKLHGEIKGAITSNSG----KVSEEDVQKMPYLKAVVMEGLRRHPPGHFVLPHAPAEDMELGGYTIPKG PLN00168
VILMYLNAGHESSGHITTWATIFLQEHPEFLQKAKEEQEQIVKRRPPAQN-GLSLKEVREMDYLSKVIDETLRLITFSLTVFR-EAKTDFSINGYIIPKG PLN02302
IVENINVAAIETTLWSIEWALAEVVNHPAVQSKVRAEINDVLGDDE-----PITESSIHKLTYLQAVIKETLRLHSPIPLLVPHMNLEEAKLGGYTIPKG PLN02394
IVVSFLLAGRDTVSTALTTLFMLLSKNPEVAAAMRAEAEAGDGGETG---AAITYEHLKGLHYTHAVLHENMRLFPPVQFDSKFCAAADVLPDGTYVGGD PLN02426
LILSLLFAGHETSSVAIALAIFFLQACPKAVEELREEHLEIARAKKELGESELNWDDYKKMDFTQCVINETLRLGNVVRFLHR-KALKDVRYKGYDIPSG PLN02500
LIILNLFVAGTDTTSTIVEWTMAELIRHPDILKHAQEELDVVVGRDR-----LLSESDLSHLTFFHAIIKETFRLHPSTPLSLPRMASEECEIAGYRIPKG PLN02687
NMIDMMIPGHDSVPVLITLAVKFLSDSPAALNLLTEENMKLKSLKELTG-EPLYWNDYLSLPFTQKVITETLRMGNVIIGVMR-KAMKDVEIKGYVIPKG PLN03141
                                                                     EXXR - motif

       510       520       530       540       550       560       570       580       590       600
....|....|....|....|....|....|....|....|....|....|....|....|....|....|....|....|....|....|....|....|
VEIFISTILVHHDSELWGDDAKEFKPERFSEG-----VLKATNGR---FSFFPFGGGPRICIAQNFALLEAKIALSMILQCFSFELSPTYTHAPTMVMTI Glycine max
VEIFISTILVHHDSELWGDDAKEFKPERFSEG-----VLKATNGR---FSFFPFGGGPRICIAQNFALLEAKIALSMILQCFSFELSPTYTHAPTMVMTI CWS5095 (G. soja)
VEIFISTILVHHDSELWGDDAKEFKPERFSEG-----VLKATNGR---FSFFPFGGGPRICIAQNFALLEAKIALSMILQCFSFELSPTYTHAPTMVMTI CW13613 (G. soja)
VEIFISTILVHHDSELWGDDAKEFKPERFSEG-----VLKATNGR---FSFFPFGGGPRICIAQNFALLEAKIALSMILQCFSFELSPTYTHAPTMVMTI CW16078 (G. soja)
LSIWIPVLAIHHSNELWGEDANEFNPERFTTR-----SFASSR------HFMPFAAGPRNCIGQTFAMMEAKIILAMLVSKFSFAISENYRHAPIVVLTI PLN02290
SRVMINAWAIGRDPNAWED-PDKFNPSRFLD----GKAPDFRGMD---FEFLPFGSGRRSCPGMQLGLYALELAVAHLLHCFNWELPHGMKPAELDMNDV PLN02183
SQVLLSRYGLGRNPKVWSD-PLSFKPERHLN---ECSEVTLTEND---LRFISFSTGKRGCAAPALGTAITTMMLARLLQGFKWKLAGSETRVELMESSH PLN02971
WKVFSSFRAVHLDPNHFKD-ARTFNPWRWQ--SNSVTTGPS-------NVFTPFGGGPRLCPGYELARVALSVFLHRLVTGFSWVPAEQDKL------VF PLN02987
TRLSVNIWAIGRDPDVWDN-PLDFTPERFFS--EKYAKINPQGND---FELIPFGAGRRICAGTRMGIVLVQYILGTLVHSFDWKLPKDV---ELNMDEV PLN00110
SKIVICIYALGRMRSVWGEDALDFKPERWISDNGGLRHEPS-------YKFMAFNSGPRTCLGKNLALLQMKMVALEIIRNYDFKVIEGHKVEPIPSILL PLN02169
WKVLPLFRNIHHSADIFSN-PGKFDPSRFEVAPKPNT-------------FMPFGNGTHSCPGNELAKLEMSIMIHHLTTKYRFGQLLERATGFSMGHSR PLN02196
WRIYVYTREINYDPFLYPD-PMTFNPWRWLEKNMESHP-----------HFMLFGGGSRMCPGKEVGTVEIATFLHYFVTQYRWEEEGNNTI------LK PLN02774
TTVNVNAWAVSRDEKEWGPNPDEFRPERFL-----EKEVDFKGTD---YEFIPFGSGRRMCPGMRLGAAMLEVPYANLLLSFNFKLPNGMKPDDINMDVM PLN02966
SHIHVCRPGLGRNPKIWKD-PLVYKPERHLQGDGITKEVTLVETE---MRFVSFSTGRRGCIGVKVGTIMMVMLLARFLQGFNWKLHQDFGPLSLEEDDA PLN03018
TIIQVNAWAVSRDTAAWGDNPNEFIPERFMN---EHKGVDFKGQD---FELLPFGSGRRMCPAMHLGIAMVEIPFANLLYKFDWSLPKGIKPEDIKMDVM PLN03234
TLVNFTVADMGMDGAAWDR-PREFLPERFMAG-GDGEGVDITGTR--EIRMMPFGAGRRICPGLGVATLHLEYFVANMVAAFEWRAAEGEAVDVDGEKLE PLN00168
WKVLVWFRTVHLDPEIYQN-PKEFNPSRWDNYTPKAG------------TFLPFGAGSRLCPGNDLAKLEISIFLHYFLLDYRLERENPECRWMFLPHTR PLN02302
SKVVVNAWWLANNPALWEN-PEEFRPERFLEKESGVDATVAGKVD---FRFLPFGVGRRSCPGIILALPILALIVGKLVRSFEMVPPPGVEKLDVSEKGG PLN02394
ARVMYHPYAMGRMPHIWGADYAAFRPARWLTGPGASFVPANP------YKYPVFQAGQRVCLGKELAVTEMKAASVAVVRAFDVEVVGENGRSGGAAAAP PLN02426
WKVLPVISAVHLDNSRYDQ-PNLFNPWRWQQQNNGASSSGSGSFSTWGNNYMPFGGGPRLCAGSELAKLEMAVFIHHLVLKFNWELAEDDKP------FA PLN02500
AELLVNVWGIARDPAIWPD-PLEYKPSRFLPG-GTHTDVDVKGND---FGLIPFGAGRRICAGLSWGLRMVTMTAATLVHAFDWQLPADQTPDKLNMDEA PLN02687
WCFLAYLRSVHLDKLYYES-PYKFNPWRWQERDMNTS------------SFSPFGGGQRLCPGLDLARLETSVFLHHLVTRFRWI-AEEDTI------IN PLN03141
```

```
              610        620        630        640
....|....|....|....|....|....|....|....|
QP---QYGAPVILHKVEKYE--------------------          Glycine max
QP---QYGAPVILHKVEKYE--------------------          CWS5095 (G. soja)
QP---QYGAPVILHKVEKYE--------------------          CW13613 (G. soja)
QP---QYGAPVILHKVEKYE--------------------          CW16078 (G. soja)
KP---KYGVQLVLKPLDL----------------------          PLN02290
FGLTAPRAVRLVAVPTYRLNCPL-----------------          PLN02183
D---MFLSKPLVLVGELRLSEDLYPMVK------------          PLN02971
FPTTRTQKRYPIFVKRRDFAT-------------------          PLN02987
FGLALQKAVPLSAMVTPRLEPNAY-LA-------------          PLN00110
RMKHGLKVTVTKKI--------------------------          PLN02169
FPKTDCPLCWPGSRRSKCRMTELPLAFLYLERGD------          PLN02196
FPRVEAPNGLHIRVQDY-----------------------          PLN02774
TGLAMHKSQHLKLVPEKVNKY-------------------          PLN02966
S---LLMAKPLHLSVEPRLAPNLYPKFRP-----------          PLN03018
TGLAMHKKEHLVLAPTKHI---------------------          PLN03234
FTVVMEKPLRARLLPRAVTV--------------------          PLN00168
PTDNCVARIKKVSSTSV-----------------------          PLN02302
QFSLHIAKHSVVAFHPISA---------------------          PLN02394
RFVPGLTASISGGLQVRVRRRVHT----------------          PLN02426
FPFVDFPNGLPIRVSRIL----------------------          PLN02500
FTLLLQRAEPLVVHPVPRLLPSAYNIA-------------          PLN02687
FPTVHMKNKLPIWIKRI-----------------------          PLN03141
```

CYP450 superfamily	GenBank accession number	Source
PLN02290	NP_176882	*Arabidopsis thaliana*
PLN02183	XP_002313762	*Populus trichocarpa*
PLN02971	NP_179820	*Arabidopsis thaliana*
PLN02987	NP_196188	*Arabidopsis thaliana*
PLN00110	XP_002314003	*Populus trichocarpa*
PLN02169	NP_176086	*Arabidopsis thaliana*
PLN02196	NP_974574	*Arabidopsis thaliana*
PLN02774	NP_001050623	*Oryza sativa*
PLN02966	XP_002868339	*Arabidopsis lyrata*
PLN03018	NP_563995	*Arabidopsis thaliana*
PLN03234	XP_002867299	*Arabidopsis lyrata*
PLN00168	NP_001064139	*Oryza sativa*
PLN02302	XP_002321248	*Populus trichocarpa*
PLN02394	NP_001046814	*Oryza sativa*
PLN02426	NP_001065766	*Oryza sativa*
PLN02500	NP_190635	*Arabidopsis thaliana*
PLN02687	NP_001064333	*Oryza sativa*
PLN03141	NP_566462	*Arabidopsis thaliana*
